# Supplementary material for: Identification of photoperiod-induced specific miRNAs in the adrenal glands of Sunite sheep (Ovis aries)
Source: Front Vet Sci. 2022 Jul 22;9:888207. doi: 10.3389/fvets.2022.888207 (PMC9354845; doi:10.3389/fvets.2022.888207)
Supplement: Supplementary file 1 [file Data_Sheet_1.ZIP › Supplementary file/miRDeep2 file.zip!/miRDeep2 file/Novel_Structure/LP42A3/Novel_671.pdf]

Provisional ID : NC_019481.2_23306
Score total : 1752.2
Score for star read(s) : 3.9
Score for read counts : 1744.7
Score for mfe : 2
Score for randfold : 1.6
Score for cons. seed :
Total read count : 3434
Mature read count : 3358
Loop read count : 0
Star read count : 76

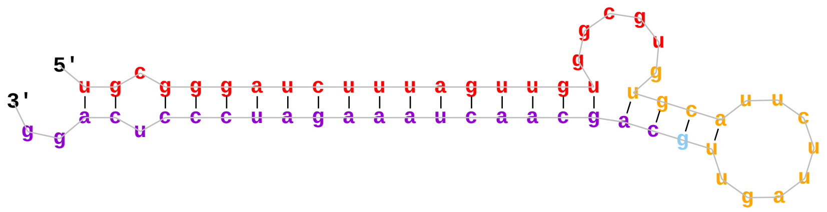

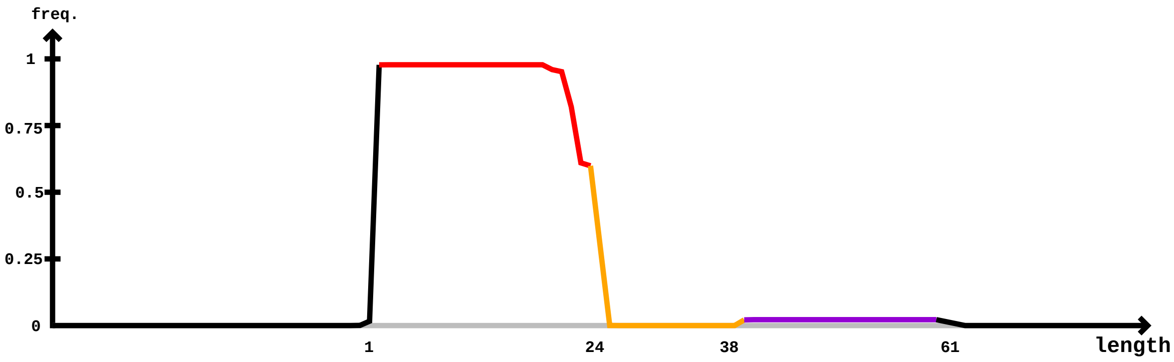


```
                                  Mature                                Star
5'- aauaacccauaaaaagcagccgacguugcggcaugcgggaucuuuaguuguggcgugugcauucuuaguugcagcaacuaaagaucccucaggcugcaaugaagaucagagau  -3'  obs
    aauaacccauaaaaagcagccgacguugcggcaugcgggaucuuuaguuguggcgugugcauucuuaguugcagcaacuaaagaucccucaggcugcaaugaagaucagagau       exp
    ....................(..((((((((.((.(((((((((((((((.......((((........)))))))))))))))))).)).)))))))))..)..........  reads  mm  sample
    ...............................caugcgggaucuuuaguuguggU...........................................................  1      1   seq
    ...............................caugcgggaucuuuaguuguggcAu.........................................................  1      1   seq
    ................................augcgggaucuuuaguugu..............................................................  3      0   seq
    ................................augcgggaucuuuaguuguA.............................................................  2      1   seq
    ................................augcgggaucuuuaguugugU............................................................  2      1   seq
    ................................augcgggaucuuuaguugugg............................................................  13     0   seq
    ................................auUcgggaucuuuaguugugg............................................................  1      1   seq
    ................................augcgggaucuuuaguuguggU...........................................................  21     1   seq
    ................................auAcgggaucuuuaguuguggc...........................................................  1      1   seq
    ................................augcgggaucuuuaguuguggcA..........................................................  10     1   seq
    ................................augcgggaucuuuagCuguggcgu.........................................................  1      1   seq
    .................................ugcgggaucuuuaguugA..............................................................  2      1   seq
    .................................ugcgggaucuuuaguugC..............................................................  4      1   seq
    .................................ugcAggaucuuuaguugu..............................................................  2      1   seq
    .................................ugcgggaucuuuaguugu..............................................................  51     0   seq
    .................................uAcgggaucuuuaguugug.............................................................  3      1   seq
    .................................ugcgggaucuuuaguuguA.............................................................  22     1   seq
    .................................ugcgggaucuuuagCugugg............................................................  1      1   seq
    .................................ugcAggaucuuuaguugugg............................................................  3      1   seq
    .................................ugcgggaucuuuaguugugA............................................................  122    1   seq
    .................................ugcgggaucuuuaguugCgg............................................................  1      1   seq
    .................................ugcgggaucuuuaguuguAg............................................................  1      1   seq
    .................................ugcgggaucuuuaguugugg............................................................  277    0   seq
    .................................ugcgggaucuuuaguugugU............................................................  32     1   seq
    .................................ugcgggaucuuuaguugugAc...........................................................  3      1   seq
    .................................ugcgggaucuuuagCuguggc...........................................................  1      1   seq
    .................................uAcgggaucuuuaguuguggc...........................................................  5      1   seq
    .................................ugcAggaucuuuaguuguggc...........................................................  7      1   seq
    .................................ugcgggaucuuuaguuguggc...........................................................  681    0   seq
    .................................ugcgggaucuuuagCuguggcg..........................................................  8      1   seq
    .................................uAcgggaucuuuaguuguggcg..........................................................  9      1   seq
    .................................ugcUggaucuuuaguuguggcg..........................................................  2      1   seq
    .................................ugcgggGucuuuaguuguggcg..........................................................  9      1   seq
```

```
                                Mature                                Star
aauaacccauaaaagcagccgacguugcggcaugcgggaucuuuaguuguggcgugugcauucuuaguugcagcaacuaaagaucccucaggcugcaaugaagaucagagau
................................ugcAggaucuuuaguuguggcgu.........................................................    21    1    seq
................................ugcgggGucuuuaguuguggcgu.........................................................    5     1    seq
................................ugcgggaucuuuagCuguggcgu.........................................................    4     1    seq
................................ugcgggaucuuuaguuguggcgu.........................................................    2011  0    seq
................................uAcgggaucuuuaguuguggcgu.........................................................    13    1    seq
................................ugcgggaucuuuaguuguggcAugu.......................................................    1     1    seq
.................................gcgggaucuuuaguuguggc...........................................................    1     0    seq
.....................................................................cagcaacuaaagaucccucag.....................    15    0    seq
.....................................................................cagcaacuaaagaucccucagg....................    58    0    seq
......................................................................agcaacuaaagaucccucagg....................    3     0    seq
```
